# Adjuvant treatment in colorectal cancer

**DOI:** 10.1038/sj.bjc.6600280

**Published:** 2002-05-06

**Authors:** B G Taal, H van Tinteren, L van t Veer

**Affiliations:** The Netherlands Cancer Institute, Antoni van Leeuwenhoek Hospital, Plesmanlaan 121, 1066 CX Amsterdam, The Netherlands

## Abstract

*British Journal of Cancer* (2002) **86**, 1525–1526. DOI: 10.1038/sj/bjc/6600280 www.bjcancer.com

© 2002 Cancer Research UK

## Sir

Iacopetta *et al* draw attention to a very important question: which patients will benefit the most from adjuvant therapy in colorectal cancer (Maughan, 2001). Earlier, they reported on a retrospective, non-randomised study of surgery with or without 5FU and Levamisole among 656 patients in Western-Australia, a treatment interaction with gender, localisation of the tumour and microsatellite instability (MSI) (Elsaleh *et al*, 2000). By extending their series, up to 891 stage III colorectal cancer patients, similar results were found with p53 in addition (Elsaleh *et al*, 2001). Chemotherapy provided maximal benefit in female patients and p53 normal tumours.

In the Dutch NACCP trial a beneficial effect of adjuvant treatment was found in stage II and III (Taal *et al*, 2001). However, as the NACCP trial was stopped early we did not reach enough statistical power to perform reliable subgroup analysis. Female patients seemed to benefit more than male, but confidence intervals were wide and the result was not statistically significant. Likewise adjuvant therapy appeared not to be of benefit in rectal cancer, but again, no significant interaction of treatment and tumour site was found and further exploration is warranted.

**Figure 1:**
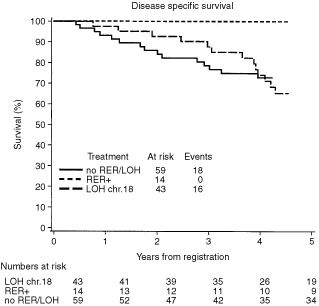

Like others (Watanabe *et al*, 2001) we have explored the role of molecular markers to select patients who might benefit most from adjuvant treatment. In a random sample of 116 patients (64 male, 53 female) in either stage II (*n*=57) or stage III (*n*=59), colonic cancer (*n*=82) as well as rectal cancer (*n*=34) in fourteen patients (12%) MSI was found and in 43 cases LOH chr18 (37%). MSI was almost exclusively found in colon cancer (*n*=13) and only once in rectal cancer, whereas LOH chr18 was present in two-thirds of colon cancer (*n*=28), but also in rectal cancer patients (*n*=15). Difference in gender and stage were not apparent. Disease specific survival revealed a superb result for the MSI positive group (100% at 5 years); in the LOH chr18 group survival was worse, but clearly better than in the group without molecular markers (Figure 1

). Subdividing the groups according to treatment makes no sense due to the small number of patients.

In conclusion, our preliminary data of molecular markers do show a major potential role for MSI instability as prognostic marker, but await inclusion of more patients.
